# HIV-Associated Lymphomas: Progress and New Challenges

**DOI:** 10.3390/jcm11051447

**Published:** 2022-03-07

**Authors:** Georgios N. Pongas, Juan C. Ramos

**Affiliations:** Department of Medicine, Division of Hematology, Sylvester Comprehensive Cancer Center, University of Miami Miller School of Medicine, Miami, FL 33136, USA; gpongas@med.miami.edu

**Keywords:** lymphoma, HIV, AIDS

## Abstract

The association of human immunodeficiency virus (HIV) and aggressive lymphomas was first reported in 1982. Before the development of effective HIV antiviral therapy, the incidence and the mortality of these lymphomas was high, with patients frequently succumbing to the disease. More lately, the combination of cART with chemoimmunotherapy significantly improved the survival outcome of the HIV-lymphomas. In this review, we discuss on describing the incidence of HIV-associated lymphomas, their clinical features, and the latest advances in the management of the various lymphoma subtypes.

## 1. Introduction

The association of human immunodeficiency virus (HIV) and aggressive lymphomas was first reported in 1982 [1]. Subsequently, systemic lymphomas with high grade or intermediate grade large-cell type occurring in HIV positive patients were included in the definition of the acquired immune deficiency syndrome (AIDS) [2]. Before the development of effective HIV antiviral therapy, the incidence and the mortality of these lymphomas was high, with patients frequently succumbing to the disease. Although AIDS-related lymphomas (ARLs) remain today amongst the most common malignancies in patients with HIV, their incidence decreased significantly after 1996 with the introduction of combination antiretroviral therapy (cART) [3,4]. More recently, the combination of cART with chemoimmunotherapy significantly improved the survival outcome of the HIV-lymphomas. In this review, we discuss the incidence of HIV-associated lymphomas, their clinical features, and the latest advances in the management of the various lymphoma subtypes.

## 2. Incidence of HIV-Associated Lymphomas

Based on cancer statistics report from 2020, NHL remains the seventh most prevalent malignancy in both sexes and ninth leading cause of cancer-related mortality [5]. The incidence of NHLs increased globally by 39% between 2007 and 2017 due to changes in population age and growth [6]. In fact, it is estimated that 1 in 108 men and 1 in 162 women can develop NHL during their lifetime [6]. For persons living with HIV (PLWH), the standardized incidence ratio (SIR) is estimated to be 23–353 higher compared with the non-HIV population [7]. Notably, the increased incidence rate of lymphoma among PLWH that was observed by the 1980s was stabilized in the 1990s and had a slight decline at the end of the decade, likely due to a decrease in AIDS incidence [8].

Diffuse large B cell lymphoma (DLBCL), Burkitt lymphoma (BL), plasmablastic lymphoma (PBL), primary DLBCL of the central nervous system (PCNS), and primary effusion lymphomas (PEL) represent the high grade B-cell lymphomas observed in PLWH, with the DLBCL and Burkitt lymphoma constituting more than 60% of the HIV-related lymphomas [9]. On the contrary, low grade B cell lymphomas such as follicular lymphoma (FL) and small lymphocytic lymphoma (SLL), and T cell lymphomas including cutaneous T-cell lymphomas (CTCL) and peripheral T-cell lymphomas (PTCL), are less frequent with a relative risk (RR) of around 15 [10]. Finally, the incidence of Hodgkin lymphoma (HL) among PLWH is approximately 7–15 higher compared with the non-HIV population [4,11]. In contrast with NHL, the incidence of HL increased following the introduction of the cART but has currently stabilized [12,13].

## 3. Diffuse Large B Cell Lymphoma

The treatment of HIV-DLBCL has evolved over the last 35 years, with significant improvement in the survival outcomes. Before the introduction of cART, patients with ARLs had a poor outcome, with median overall survival (OS) of 6 months mainly due to infections and chemotherapy failure. That led to the hypothesis that less dose-intense regimens may be preferable for treatment of HIV-lymphomas. However, a trial comparing standard dose m-BACOD (methotrexate, bleomycin, doxorubicin, cyclophosphamide, vincristine, and dexamethasone) with GM-CSF support vs. low-dose m-BACOD without GM-CSF did not show any survival advantage, casting doubts on this hypothesis [14].

Following the introduction of cART, the survival outcome of HIV-lymphomas improved dramatically. Notably, an analysis in 485 patients with HIV-lymphomas treated with risk-adapted intensive chemotherapy showed that cART and not the intensity of CHOP-like chemotherapy predicted the patient’s survival [15]. This observation likely stemmed from the improved immunosurveillance and the decreased incidence of infections.

Various combination chemotherapies were initially studied in HIV-DLBCL including the ACVB (doxorubicin, cyclophosphamide, vindesine, bleomycin, and prednisolone), CDE (cyclophosphamide, doxorubicin, and etoposide), and CHOP regimen (cyclophosphamide, doxorubicin, vincristine, and prednisone) [16,17]. In 2003, Little et al. reported that dose-adjusted EPOCH (etoposide, prednisone, vincristine, cyclophosphamide, and doxorubicin) could achieve 74% complete response (CR) in patients with aggressive HIV-lymphomas with a 53-month OS of 79% [18]. These results were superior compared with the historic outcomes of CHOP, which demonstrated 33% CR with 36-month OS of 25% [19]. To build on EPOCH, the AIDS Malignancy Consortium (AMC) evaluated the addition of rituximab concurrently to six cycles of EPOCH compared with EPOCH up to six cycles followed by sequential six doses of rituximab [20]. The study showed superior CR 73% for the concurrent arm compared with 55% for the sequential arm, without any differences in the OS, and rejected the null hypothesis of 50% CR associated with CHOP with or without rituximab [20]. It also demonstrated a comparable toxicity profile among the two arms, with grade 3 or 4 adverse events in at least 5% of patients [20]. The superiority of EPOCH-R was also demonstrated in a pooled analysis from prospective phase II and III clinical trials analyzing the outcome of 1546 patients between 1990 until 2010, which showed a significant improvement in CR and OS with EPOCH-R over R-CHOP [21]. Therefore, EPOCH-R is currently listed as the preferred regimen for HIV-DLBCL under the National Comprehensive Cancer Network (NCCN) guidelines [22]. While the cycles of chemotherapy for management of HIV-associated DLBCL is following the same paradigm as DLBCL in the non-HIV population, a prospective clinical trial in HIV-DLBCL and high-grade lymphoma demonstrated that a response-adapted strategy by a CT may allow a shorter duration of chemotherapy of four cycles for patients achieving a CR after the second cycle [23].

In addition to immunotherapies, the addition of epigenic agents that could disrupt the viral latency and induce an oncolytic effect is an interesting therapeutic concept. AMC recently published a sequential phase 2 randomized trial combining EPOCH (+/− rituximab based on the CD20 expression) with and without vorinostat in 90 patients with aggressive HIV-NHL [24]. The trial stemmed from a preclinical notion of the synergism between the histone deacetylase inhibitor (HDAC) vorinostat with etoposide, anthracycline, and rituximab and the ability of HDAC inhibitors to disrupt viral latency [25,26,27,28]. The trial allowed pre-protocol treatment in order to capture the high-risk patients who could not wait for the randomization. The CR rates were 74% vs. 68% for the R-EPOCH vs. R-EPOCH with vorinostat, demonstrating no benefit from the addition of the HDAC inhibitor in the upfront setting when given on a short (5-day) schedule. Notably, patients with Myc+ DLBCL by protein expression had a significantly inferior 3-year event-free survival (EFS) of 44% as compared with 83% in Myc- DLBCL patients. Therefore, these results highlight the importance of the Myc protein expression status in the prognostication of HIV-DLBCL lymphomas treated with EPOCH-based chemotherapy and pave the way for investigation of future drug combinations targeting Myc+ HIV-DLBCL. While the addition of an HDAC inhibitor did not lead to improvement in the survival outcome, the potent preclinical data are intriguing enough to question whether such a combined approach could have a role in the relapsed setting. Oncolysis is induced through viral replication and immune-mediated cell death of latently infected cancer cells by EBV or the human herpesvirus 8 (HHV8) [29,30,31,32,33]. There currently are two early phase clinical trials using lytic-inducing HDAC inhibitors for relapsed/refractory EBV+ lymphomas that are actively enrolling patients with HIV: (1) A trial of nanatinostat in combination with valganciclovir (NCT05011058) and (2) a dose expansion/escalation trial of oral VRx-3996 and valganciclovir (NCT03397706). Perhaps disruption of viral latency in combination with newer immunostimulatory strategies such as immune checkpoint inhibitors PDL1 monoclonal antibodies or CD47 blocking agents will lead to the immune-mediated death of cancer cells latently affected by EBV or KSHV and should be explored in future clinical trials.

### Role of CNS Prophylaxis in HIV DLBCL

CNS prophylaxis in HIV-DLBCL is recommended by the NCCN guidelines [34]. Since early relapses in HIV-lymphoma are more common, obtaining a flow cytometry is of paramount significance to avoid underdiagnosis with the conventional cytology [35]. Notably, a retrospective study evaluating factors and outcomes associated with CNS involvement at baseline and relapse in 886 patients with newly diagnosed HIV-lymphoma did not identify any significant association between age-adjusted IPI, LDH, number of extranodal sites, and risk of CNS relapse [35]. Additionally, intrathecal CNS prophylaxis with one vs. three agents did not impact the risk for CNS relapse of HIV-lymphoma, and despite the prophylaxis, 5% of patients experienced CNS relapse, resembling the incidence of CNS relapse in the regular population [35,36].

## 4. HIV-Associated Burkitt Lymphoma

BL comprises 20% to 40% of the aggressive HIV-lymphomas [37,38]. In contrast with DLBCL, the incidence of BL is lowest among people with ≤50 CD4 lymphocytes/ul vs. those with ≥250 lymphocytes/ul [39]. Notably, in a large retrospective study of 712 patients with HIV and NHL, the sole key predictor for BL was the cumulative HIV RNA and not the CD4, implying that immunosurveillance is not a meaningful control for HIV-associated BL [40].

Similar to DLBCL in the pre-ART era, intensive chemotherapy regimens were initially avoided to mitigate events of treatment-related mortality. In 2003, a retrospective study of cyclophosphamide, vincristine, doxorubicin, and methotrexate/ifosfamide, etoposide, and cytarabine (CODOX-M/IVAC) with ART in HIV-BL showed a CR of 63% with 2-year EFS of 60% and similar rates of myelosuppression and infectious complications with the HIV-negative patients. To build on the CODOX-M/IVAC backbone, the AMC 048 trial added rituximab, reduced the cyclophosphamide, rescheduled the methotrexate, and used a combination of intrathecal chemotherapy [41]. Impressively, the modified CODOX-M/IVAC-R showed a two-year OS of 69%, with favorable toxicity compared with the parent regimen [41].

In 2013, an uncontrolled prospective study from the National Cancer Institute (NCI) compared the low intensity EPOCH-R regimens, DA-EPOCH-R in HIV negative patients, and a short course regimen with a double dose of rituximab (SC-EPOCH-R) in HIV positive patients [42]. With a median follow up of 73 months in the SC-EPOCH-R group and 86 months in the DA-EPOCH-R group, the rates of progression-free survival (PFS) and OS were, respectively, 100% and 90% with SC-EPOCH-R and 95% and 100% with DA-EPOCH-R [42]. These results demonstrated that a low-intensity EPOCH-R-based regimen can be highly effective in HIV patients with BL [42]. To build on these results, a subsequent multicenter risk-adapted study of DA-EPOCH-R in BL studied three cycles in low-risk patients without CNS prophylaxis and six cycles with intrathecal CNS prophylaxis for the high risk with extended intrathecal treatment for patients with leptomeningeal enhancement [43]. This risk-adapted strategy of DA-EPOCH-R was effective regardless of HIV status. At a median follow-up of 58.7 months, EFS and OS were respectively 84.5% and 87.0% for the low-risk patients and 100% and 82.1% for the high-risk patients [43].

### Role of CNS Prophylaxis in HIV-BL

Given the high risk of CNS relapse in patients with BL, CNS prophylaxis is recommended. Prophylactic chemotherapy can be given with intrathecal chemotherapy or intravenously with high dose cytarabine or high dose methotrexate [41,43]. For patients with low-risk HIV BL based on normal LDH, ECOG performance status ≤ 1, stage ≤ 2, and tumor size < 7 cm, who are treated with the risk-adapted approach of DA-EPOCH-R, no prophylactic chemotherapy is needed, whereas the high-risk patients require IT methotrexate with each cycle [43]. With this practice, relapses in the CNS are uncommon, with only 2% relapses in the CNS parenchyma [43].

## 5. Primary Effusion Lymphoma

PEL is an aggressive B-cell NHL that was originally described in patients with AIDS. It comprises approximately 1% to 5% of ARLs [44]. It is characterized by development of lymphomatous effusions in the pleural, pericardial, or abdominal cavities. The tumor is classically infected by the HHV8, which is the oncogenic driver, while most cases are also co-infected by EBV [45,46]. The management of PEL is with chemotherapy, albeit there is no universally accepted regimen that defines the standard of care. CHOP-like regimens can achieve 41% to 62% complete responses, with a median survival of 10.2 months [47]. In a retrospective study from the NCI, modified EPOCH resulted in 3 years cancer-specific survival of 47%, with no patient who survived 2 years after the diagnosis of PEL dying of PEL [48]. In AMC-075 trial, five of seven (71% patients) with PEL treated with EPOCH-based chemotherapy achieved a CR and remained disease-free over 2 years [49]. Based on preclinical data demonstrating a cytotoxic effect of lenalidomide in PEL cell lines, a study combining lenalidomide with R-EPOCH is currently active (NCT02911142). While there are no prospective studies evaluating the role of cART in PEL, the median survival of patients with PEL has improved since the introduction of cART, and hence, the use of cART during chemotherapy is recommended.

## 6. Plasmablastic Lymphoma

PBL is a distinct subtype of B-cell lymphoma more commonly seen in patients with HIV, where co-infection with EBV is usually the case [49]. Extranodal involvement can present in 95% of patients, with 48% involving the oral cavity and jaw [50]. Notably, more than 65% of HIV patients present with advanced clinical stage [50]. The histologic diagnosis of PBL can be challenging because it has cytomorphologic features of both lymphoma and plasmacytic neoplasms. No standard of care is widely accepted for the management of PBL. Due to CD20 negativity and poor survival outcome with CHOP, EPOCH is recommended by experts, often in combination with the proteasome inhibitor bortezomib. A retrospective study of 16 patients with PBL treated with EPOCH plus bortezomib, 38% of whom had HIV, showed a complete response in 94% of the patients, with median OS of 62 months [51]. So far, AMC-075 has had the largest number of patients with PBL treated with EPOCH-based chemotherapy under a single clinical trial (*n* = 15) with 10/15 (67%) collectively achieving a CR and 60% event-free survival at 3 years [49].

Two studies, currently in clinical trials evaluating the addition of the anti-CD38 daratumumab to EPOCH (NCT04139304) and the belantamab mafodotin (NCT04676360), a B-cell maturation antigen (BCMA) conjugated with the cytotoxic agent maleimidocaproyl monomethyl auristatin F (mcMMAF), will provide additional information about the management of PBL.

## 7. Primary CNS Lymphoma

The incidence of HIV-associated primary central nervous system lymphoma (HIV-PCNS) decreased significantly since the introduction of ART. EBV is identified in almost all the HIV-PCNS and is considered the driver of these lymphomas [52]. The presence of EBV DNA in the CSF can also assist in the diagnosis of PCNS when a biopsy is not feasible [53]. Before the introduction of ART, treatment of HIV-PCNS was based on palliative whole brain radiation, with poor response rates and median OS (mOS) of 3 months [54]. However, increasing evidence showed that high-dose methotrexate combined with ART could be administered in HIV-PCNS with curative intent [55]. In the first prospective study of radiation-sparing therapy in HIV-PCNS, the combination of HD-MTX with ART and rituximab achieved a 60-month OS of 67%, and all participants with sustained CR were able to live on their own unassisted and free of severe general neurocognitive dysfunction [56].

## 8. Current Challenges and Future Directions in HIV-Associated Lymphoid Malignancies

Despite the introduction of cART and the advances in treatment of HIV-lymphomas, the survival outcome of the relapsed and refractory lymphomas remains dismal. The development of new immunotherapies and the exploration of chimeric antigen receptor T (CAR-T) cellular therapies is an area of unmet need.

### 8.1. Autologous and Allogeneic Stem Cell Transplantation for HIV-Associated Lymphomas

Relapsed HIV-lymphomas are associated with poor outcome. In a large retrospective study of 254 patients who achieved a CR with the first chemotherapy, the relapse rate was 11% and was equally observed among DLBCL, BL, and PBLs [57]. Notably, similar relapse rates were observed in the general non-HIV population. Since the introduction of cART, high dose-intensive therapy (HDT) and peripheral stem cell transplantation (PBSCT) became feasible for HIV-lymphomas. In a prospective study of HDT and PBSCT in 50 patients with HIV-lymphoma, the mOS was 33 months, with 42% of patients being alive and disease-free after 44 months [58]. Two additional retrospective studies demonstrated that the outcome of autologous stem cell transplantation (ASCT) for NHL and HL was similar regardless of HIV status [59,60]. A subsequent trial of the Blood and Marrow Transplant Clinical Trials Network (BMT CTN) 0803/(AMC-071) in patients with HIV-lymphoma demonstrated a 2-year OS and PFS of 82% and 80%, respectively [61]. This study concluded that patients with HIV-lymphoma should be considered for ASCT when they meet the standard transplant criteria [61,62].

Given the curative potential of allogeneic transplant (AlloHCT) for lymphoid malignancies, the BMT CTN/AMC performed a prospective study to evaluate the safety and effectiveness of AlloHCT in 17 patients with HIV-lymphoma [62]. Notably, there was no 100-day nonrelapse mortality (NRM), and the incidence of grade I and IV acute graft-versus-host disease (GVHD) was 41% in the first 100 days. The OS was 59% in the first one year, with 63% of deaths due to relapse and progression of the lymphoma [62].

### 8.2. Chimeric Antigen T Cells against CD19 and HIV

The first two cases of HIV-lymphoma treated with the commercially available anti-CD19 chimeric antigen T cells (CAR-T) axicabtagene ciloleucel were reported in 2019. This report highlighted the fact that CAR-T production can be manufactured in HIV patients receiving cART with CD4 as low as 52 cells/mm^3^; the CAR-T is safe with concurrent administration of cART; and the CAR-T can achieve long lasting effects [63]. In a subsequent retrospective study of 10 patients with HIV-DLBCL, 80% achieved a CR, including two patients with prior CNS involvement. Notably, only 20% of patients experienced grade ≥ 2 cytokine release syndrome (CRS), demonstrating the efficacy of the CAR-T therapies with manageable toxicity [64].

While utilization of CAR-T against CD19 for treatment of HIV-DLBCL is still emerging, the concept of CAR-T development against HIV was introduced approximately two decades ago. CAR-T bearing a chimeric T-cell receptor targeting the HIV glycoprotein gp120 failed initially to demonstrate differences in viral burden, albeit a decade-long follow-up demonstrated the longevity of these cells [65,66]. Notably the development of CAR-T against multiple highly conserved sites of the HIV-1 envelope glycoprotein, termed duoCAR-T, was able to reduce by 97% the cellular HIV infection [67]. These results suggest that multispecific duoCAR-T may be an effective approach for the treatment of patients with HIV infection, obviating the need for long term c-ART. Manufacturing of HIV-specific T cells (HSTs) against HIV epitopes less prone to escape mutations broadens further the applicability of cellular therapies for the HIV suppression and potentially its clearance [68]. Clinical trials with duo CAR-T and HST are underway, hoping to translate the impressive experimental findings to the curative outcome of HIV.

#### Bispecific Antibodies against HIV-Related Lymphomas

Bispecific antibodies (BsAbs) are designed to bind to two different epitopes. Most BsAbs are developed to bind targets on different cells. In immunotherapy, BsAbs can improve tumor eradication by bringing cytotoxic T cells or natural Killer (NK)-cells to bear against the tumor cells. Various BsAbs have been developed against targets expressed in lymphoma cells, such as CD19 or CD20, which are currently in clinical development. Abstracts presented in the annual conference of American Society of Hematology (ASH) 2020 showed remarkable results, with complete responses between 30% and 50% in heavily pretreated relapsed and refractory B-cell DLBCL [69,70]. To the best of our knowledge, BsAbs have not been explored in patients with HIV-lymphomas, and some studies excluded HIV-infected patients. Future studies should explore the role of BsAbs in combination with ARTs in HIV-related lymphomas.

## 9. COVID-19 Vaccines and Monoclonal Antibodies for HIV-Infected Patients

People with HIV can receive COVID-19 vaccines regardless of the CD4 T cell count based on the COVID-19 treatment guidelines panel (the Panel) provided by the National Institutes of health (NIH) [71]. Guidelines from the Centers for Disease Control and Prevention (CDC) and National Comprehensive Cancer Network (NCCN) recommend a third dose of the mRNA vaccines for patients with advanced or untreated HIV infection [71].

The NCCN recommend the use of tixagevimab and cilgavimab for pre-exposure prophylaxis in patients with advanced or untreated HIV infection [71]. While the two anti-SARS-CoV2-monoclonal antibodies (mAbs) combinations bamlanivimab plus etesevimab and casirivimab plus imdevimab have received Food and Drug Administration (FDA) for post-exposure prophylaxis, the Panel recommends against their use in patients with HIV because the omicron variant is the currently dominant variant in the United States and is not susceptible to these mAbs [71].

## 10. Conclusions

Since the introduction of cART, the life expectancy of PLWH has increased and is approaching that of the general population. While combination of frontline chemo-immunotherapy with cART has resulted in a remarkable prolongation of the overall survival among patients with ARLs, the management of the relapsed and refractory disease remains a challenge and an area of unmet need. While ASCT can cure a small portion of these patients, a significant percentage will eventually succumb to their disease. Future directions include the optimization and further development of epigenetic therapies exploiting the vulnerabilities of the EBV and HHV8 viruses that are implicated in the pathogenesis of HIV-lymphomas, cellular therapies such as chimeric antigen T cells, and also bispecific antibodies targeting viral antigens or antigens expressed in the lymphoma cells.
